# Optimizing hyperbaric oxygen initiation time in carbon monoxide poisoning: a 3-hour window enhances neurological recovery via lactate clearance

**DOI:** 10.1515/med-2025-1351

**Published:** 2026-01-13

**Authors:** Dongjun Xu, Xiaoqin Xu, Hui Sun, Jun Xu, Danting Fei, Yaye Shen

**Affiliations:** Department of Emergency, Affiliated Hospital of Jiaxing University, The First Hospital of Jiaxing, Jiaxing, Zhejiang Province, China

**Keywords:** acute carbon monoxide poisoning, hyperbaric oxygen therapy, multiple organ dysfunction, delayed encephalopathy, diagnosis, prognosis

## Abstract

**Objectives:**

To explore the optimal initiation time of hyperbaric oxygen therapy (HBOT) for moderate-to-severe acute carbon monoxide poisoning (ACOP) and to assess the prognostic value of lactate clearance and early MRI findings.

**Methods:**

This single-center retrospective study included 12 ACOP patients (2020–2023) treated with HBOT within 6 h after admission. Patients were categorized by HBOT initiation time: early (≤3 h, n=8) and delayed (>3 h, n=4). Clinical, biochemical, and imaging data were analyzed. Primary outcomes were time to regain consciousness and Barthel Index at 6 months.

**Results:**

Median HBOT initiation was 112 min. Early treatment was associated with faster organ function recovery (greater SOFA score reduction, p<0.05). Lactate normalized within a median of 17.5 h, and clearance >50 % in 24 h correlated with better neurological outcomes (p=0.002). MRI detected early globus pallidus injury more sensitively than CT. At 6 months, 83.3 % recovered functionally; one developed delayed encephalopathy.

**Conclusions:**

Early HBOT initiation (≤3 h) may facilitate metabolic and neurological recovery in ACOP. Early lactate clearance and MRI findings may serve as prognostic markers. Given the small sample size and absence of a non-HBOT control group, these results are exploratory and require confirmation in larger studies.

## Introduction

Carbon monoxide (CO) poisoning arises from inhalation of CO produced by incomplete combustion [1] and is a major global health concern. The annual global incidence of acute carbon monoxide poisoning (ACOP) is approximately 13.7/100,000 with >43,000 deaths [2]. China shows a higher incidence (21.8/100,000), showing urban-rural differences associated with coal heating, unsafe gas water heater use, and suicide-related exposure. ACOP can rapidly cause severe injury or death [3], and delayed encephalopathy may occur, producing long-term disability [4].

Common exposure sources include inadequate exhaust of combustion devices, operation of vehicles in confined spaces, and fires [5], 6]. Symptoms range from dizziness and headache to coma and multiple organ dysfunction [4]. Management includes life support, glucocorticoids, neuroprotective agents, and oxygen therapy. Normobaric oxygen and hyperbaric oxygen therapy (HBOT) increase arterial oxygen partial pressure to accelerate carboxyhemoglobin dissociation [7], 8]. The CO half-life decreases from 320 min (room air) to 74 min with normobaric oxygen and to 20 min with HBOT [1], and HBOT reverses CO-induced inflammation and mitochondrial dysfunction [9].

The optimal HBOT timing, treatment course, and patient selection remain unresolved. Although HBOT is recommended for moderate to severe ACOP [10], evidence regarding early (<6 h) vs. delayed HBOT remains inconsistent [11], [12], [13], [14]. Synergistic effects of HBOT with glucocorticoids and antioxidants require clarification. Prognostic markers remain limited, as the Sequential Organ Failure Assessment (SOFA) score has restricted dynamic value, and the significance of globus pallidus MRI abnormalities for long-term recovery is debated.

This study integrates clinical data from 12 moderate to severe ACOP cases to compare HBOT initiation ≤3 h vs. >3 h on consciousness recovery time and SOFA score improvement and proposes a prognostic model incorporating lactate clearance, cranial MRI features, and the Barthel Index to support optimization of HBOT strategies and individualized outcome prediction.

## Materials and methods

### Study design

Data were obtained from the electronic medical records of patients diagnosed with ACOP and admitted between January 2020 and June 2023. A three-stage screening process was applied: initial screening by ICD-10 code T58 (n=214) → manual verification (n=58) → exclusion of confounding factors, resulting in the inclusion of 12 eligible cases. Sample size was calculated using PASS 15.0 software, with α=0.05 and a power of 80 %. The estimated sample size was 10 cases per group; a total of 12 cases were ultimately included, accounting for a 15 % anticipated loss to follow-up.

### Inclusion and exclusion criteria of cases

**Figure 1:**
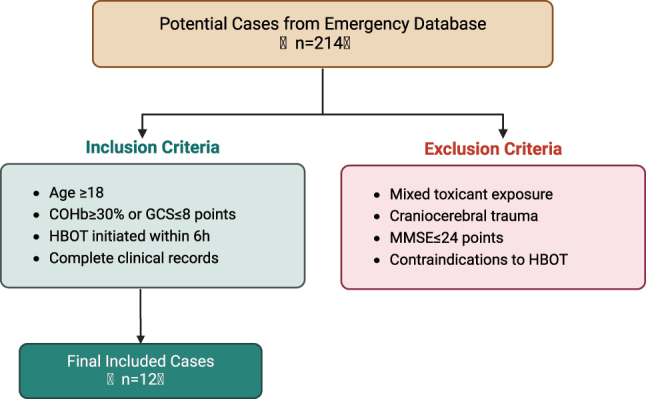
Flowchart of case inclusion and exclusion criteria.

Patients were included if they met all of the following criteria: 1. Age ≥18 years; 2. Met the criteria for moderate to severe ACOP according to the *Chinese expert consensus on the diagnosis and treatment of acute carbon monoxide poisoning (2020 Edition)* (i.e., carboxyhemoglobin (COHb) ≥30 % or Glasgow Coma Scale [GCS] score ≤8); 3. Received HBOT within 6 h of hospital admission; 4. Had complete clinical data available. Exclusion criteria were as follows: 1. Mixed substance inhalation (positive toxicology screening); 2. Concurrent traumatic brain injury (confirmed by computed tomography; CT); 3. Severe pre-existing neurological disorders (Mini-Mental State Examination [MMSE] score ≤24); 4. Contraindications to HBOT (e.g., untreated pneumothorax) (Figure 1).

### Data collection and quality control

Data were collected using a standardized case report form (CRF), including: (1) Baseline characteristics: age, sex, cause of poisoning (suicidal/accidental), exposure space volume (m^3^), and type of combustible material; (2) Laboratory parameters: carboxyhemoglobin (COHb, Radiometer ABL90 FLEX), lactate (enzymatic electrode method), liver and renal function tests (Beckman AU5800); (3) Imaging: cranial CT (GE Revolution 256-slice) and MRI (Siemens Skyra 3.0T), independently interpreted by two senior radiologists blinded to clinical data (κ=0.87); (4) Treatment parameters: HBOT initiation time (interval from emergency department arrival to chamber pressurization), treatment pressure (MPa), and pressure holding duration (min); (5) Prognostic measures: Sequential Organ Failure Assessment (SOFA) score, Barthel Index, and Modified Rankin Scale (mRS).

SOFA scores were assessed at 0 h (admission), 24 h, and 72 h by two attending ICU physicians who were blinded to HBOT initiation grouping. The Barthel Index and mRS were evaluated at discharge and at 6-month follow-up by rehabilitation physicians not involved in clinical treatment. All assessors received standardized training and performed evaluations under double-blind conditions, with patient identifiers coded and treatment allocation concealed. Data analysts were also blinded to group assignments to ensure objectivity.

### Intervention protocol

All patients received stepwise comprehensive therapy. (1) Emergency management included high-flow oxygen via face mask (FiO_2_ 100 %), establishment of intravenous access, and continuous cardiac monitoring. (2) Hyperbaric oxygen therapy was performed in a GYQ-3200/0.3 multiplace chamber (China State Shipbuilding Corporation) at a treatment pressure of 0.25 MPa for 60 min per session. Treatment frequency was adjusted according to the SOFA score: once daily for SOFA ≤4 and twice daily for SOFA >4, with an interval of at least 8 h between sessions. During HBOT, patients breathed pure oxygen via sealed masks, and vital signs were continuously monitored. (3) Organ support included goal-directed fluid resuscitation (CVP 8–12 mmHg), norepinephrine to maintain MAP ≥65 mmHg, and lung-protective ventilation strategies (tidal volume 6–8 mL/kg PBW). HBOT initiation time was defined as the interval from emergency department arrival to the start of pressurization.

### Statistical analysis

Statistical analyses were performed using SPSS version 26.0 and R version 4.2.1. Continuous variables were expressed as meana ± standard deviation and compared between groups using the Mann-Whitney U test. Categorical variables were presented as frequencies (%) and compared using Fisher’s exact test. Linear regression analysis was applied for association assessment. All statistical tests were two-tailed, and a *p*-value <0.05 was considered statistically significant.

### Ethics approval and consent to participate

This study was approved by the institutional ethics committee (Approval No.: 2024-KY-771) and conducted in accordance with the principles of the Declaration of Helsinki.

### Informed consent

All the patients have been informed and signed informed consent before the experiments.

## Results

### Demographic characteristics and poisoning etiology distribution

The study enrolled 12 moderate-to-severe ACOP patients with a median age of 41 years (interquartile range [IQR] 32–52 years), demonstrating a bimodal age distribution: 21–30 years (25.0 %, 3/12), 31–40 years (33.3 %, 4/12), 41–50 years (16.7 %, 2/12), 51–60 years (16.7 %, 2/12), and >60 years (8.3 %, 1/12). Male predominance was significant (male: female=11:1), with the sole female patient being a 58-year-old winter heating poisoning case. Further analysis revealed that suicidal poisoning cases (6/12, 50 %) clustered in 25–35 years (median 29 years, IQR 26–32 years), whereas accidental poisoning cases (6/12, 50 %) had a median age of 53 years (IQR 45–61 years), showing statistically significant intergroup difference (χ^2^=7.89, p=0.019). All poisonings occurred in enclosed spaces, with 75.0 % (9/12) caused by charcoal combustion and 25.0 % (3/12) by fire accidents. Winter incidence reached 41.7 % (5/12), and 41.7 % (5/12) patients had comorbidities (hypertension/diabetes/stroke history), consistent with national epidemiological data (Tables 1 and 2).

**Table 1:** Demographic characteristics and etiology distribution.

Category	Indicator/subgroup	Value (n=12)	Statistical results
Demographics	Age	Median (IQR): 41 years (32–52)	–
	Age distribution	21–30 years: 3 cases (25.0 %)	–
		31–40 years: 4 cases (33.3 %)	–
		41–50 years: 2 cases (16.7 %)	–
		51–60 years: 2 cases (16.7 %)	–
		>60 years: 1 case (8.3 %)	–
	Gender	Male: Female=11:1	–
Etiology	Poisoning type	Suicidal poisoning: 6 cases (50.0 %)	χ^2^=7.89, p=0.019
		Accidental poisoning: 6 cases (50.0 %)	–
	Median age (IQR)	Suicidal: 29 years (26–32)	–
		Accidental: 53 years (45–61)	–
	Poisoning source	Charcoal burning: 9 cases (75.0 %)	–
		Fire accident: 3 cases (25.0 %)	–
	Seasonal distribution	Winter: 5 cases (41.7 %)	–
Comorbidities	Presence of comorbidities	5 cases (41.7 %)	–
		Hypertension: 3 cases (25.0 %)	–
		Diabetes mellitus: 2 cases (16.7 %)	–
		Stroke: 1 case (8.3 %)	

**Table 2:** Basic information of 12 patients with carbon monoxide poisoning.

ID	Sex	Age	Cause of poisoning	Time from poisoning to hospital, h	Season of onset
Case 1	Female	66	Charcoal burning (accidental)	2.0	Spring
Case 2	Male	29	Charcoal burning (intentional)	4.0	Spring
Case 3	Male	25	Charcoal burning (intentional)	1.0	Winter
Case 4	Male	29	Fire accident (intentional)	0.5	Spring
Case 5	Male	36	Charcoal burning (intentional)	2.0	Spring
Case 6	Male	21	Charcoal burning (intentional)	5.0	Winter
Case 7	Male	49	Charcoal burning (accidental)	2.0	Summer
Case 8	Male	50	Charcoal burning (accidental)	1.0	Winter
Case 9	Male	57	Fire accident (accidental)	0.5	Winter
Case 10	Male	46	Fire accident (accidental)	0.5	Autumn
Case 11	Male	50	Charcoal burning (accidental)	6.0	Winter
Case 12	Male	29	Charcoal burning (intentional)	6.0	Autumn

### Laboratory parameters and features of multiple organ dysfunction

At admission, COHb levels, as shown in Table 3, demonstrate a marked stratification. 41.7 % (5/12) of patients had COHb levels ≥30 % (with a maximum of 50 %), while 25.0 % (3/12) had levels between 10 and 30 %, indicating significant variation in poisoning severity. For example, Case 1 presented with a COHb level of 35.1 %, loss of consciousness, and a SOFA score of 4, indicating moderate organ dysfunction. After treatment, the COHb level decreased to 2.1 %, suggesting good recovery. However, Case 2, despite having a lower COHb level (25.2 %), showed more severe clinical symptoms, a SOFA score of only 3, and slower recovery, which may be associated with delayed admission and initiation of high treatment.

**Table 3:** Summary of patient auxiliary examination results and clinical Scores.

ID	PSS	SOFA	Apatche Ⅱ	Imaging findings	Sodium/potassium, mmol/L	LDH (IU/L)	Creatine kinase (IU/L)	Lactic acid, mmol/L	PH	AST, IU/L	ALT, IU/L	Cr, µmol/L	Oxygenation index, mmHg	Admission COHb, %	Shock	Chief complaint
Case 1	2	4	19	CT+, MR+	137.9/3.62	179	66	7.3	7.31	65	61	64	183	35.1	Yes	Unconsciousness
Case 2	2	3	10	CT–, MR+	141.6/3.19	161	433	3.2	7.39	21	17	74.8	308	25.2	No	Unconsciousness
Case 3	3	5	17	CT–, MR–	140.0/4.90	167	322	5.9	7.33	30	26	99.7	287	38.8	Yes	Unconsciousness
Case 4	3	5	13	CT–, MR–	139.1/3.49	155	143	6 0.0	7.34	28	25	65.2	187	44.7	Yes	Unconsciousness
Case 5	3	5	14	CT+, MR+	136.5/3.30	403	4,938	2.4	7.51	93	64	77.2	256	2.4	No	Unconsciousness
Case 6	3	5	11	CT–, MR+	141.6/3.50	816	19,271	3	7.46	245	63	93.4	161	3	No	Unconsciousness
Case 7	3	6	22	CT–, MR+	143.1/3.57	428	5,276	17.7	7.18	88	97	153.5	335	49.5	Yes	Unconsciousness
Case 8	3	10	26	CT–	142.0/3.10	615	5,864	9	7.06	164	76	153.3	111	22.5	Yes	Unconsciousness
Case 9	3	5	16	CT–	132.0/3.54	197	199	5.2	7.25	31	80	129.7	156	38.5	No	Burns causedwidespread pain
Case 10	3	7	17	CT–, MR–	140.1/3.62	484	146	14.8	7.24	69	45	637.2	169	15.9	No	Burns causedwidespread pain
Case 11	3	9	26	CT–	157.0/3.50	668	5,407	2.5	7.22	52	35	512.4	212	1.2	No	Unconsciousness
Case 12	3	7	26	CT–	142.0/2.60	960	16,214	4.1	7.51	462	160	163.8	283	30.9	Yes	Unconsciousness

COHb, carboxyhemoglobin; PaO2/FiO2, partial arterial oxygen pressure to fraction of inspired oxygen ratio; Cr, creatinine; ALT, alanine aminotransferase; AST, aspartate aminotransferase; LDH, lactate dehydrogenase; Apatche Ⅱ, acute physiology and chronic health evaluation II; SOFA, sequential organ failure assessment; PSS, poisoning severity score; +, positive; –, negative.

All patients showed elevated lactate levels, ranging from 2.4 to 17.7 mmol/L, and 91.7 % (11/12) exhibited metabolic acidosis (pH 7.06–7.35). In particular, Case 7 had a lactate level as high as 17.7 mmol/L, indicating severe acidosis, with a SOFA score of 6, reflecting profound organ dysfunction. Although the COHb level decreased to 1.1 %, the high lactate level indicated significant multi-organ damage.

Regarding organ injury, Table 3 shows that 66.7 % (8/12) of patients had abnormal liver function (ALT 45–160 IU/L), 50.0 % (6/12) had renal impairment (creatinine 142–637 μmol/L), and 100 % of patients experienced acute lung injury (PaO_2_/FiO_2_ ratio 156–388). For example, Case 4 had a pre-treatment PaO_2_/FiO_2_ ratio of 65.2, indicating severe lung injury, and an ALT of 93 IU/L, reflecting liver damage. Following treatment, the patient’s lung function improved (PaO_2_/FiO_2_ increased to 187) and ALT decreased to 64 IU/L, indicating progressive clinical recovery.

**Figure 2:**
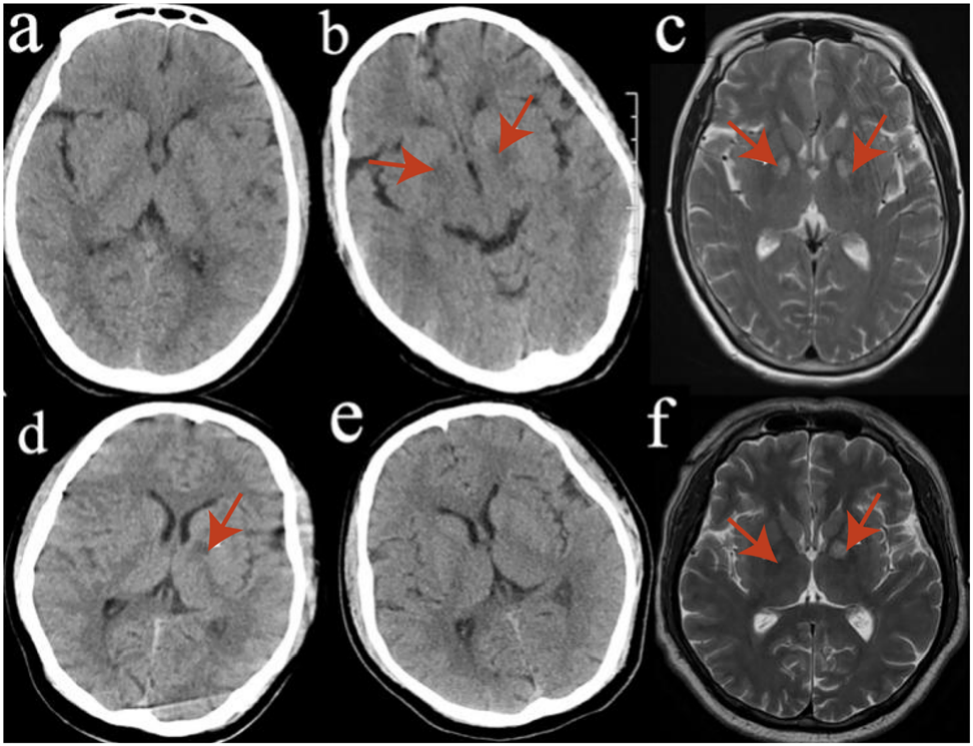
Representative cranial imaging of two patients. Note: (a) Day 1 cranial CT of case 2 showing no obvious abnormalities. (b) Day 6 cranial CT of Case 2 displaying symmetrical low-density changes in the bilateral globus pallidus (red arrows). (c) Day 2 cranial MRI of Case 2 demonstrating symmetrical hyperintensities in the bilateral globus pallidus (red arrows). (d) Day 1 cranial CT of Case 4 showing a low-density lesion in the left globus pallidus (red arrow). (e) Follow-up CT scan at 2 weeks in Case 4 showing resolution of the lesion. (f) Day 2 cranial MRI of Case 4 demonstrating bilateral globus pallidus hyperintensities (red arrows). CT: computed tomography; MRI: Magnetic resonance imaging.

All 12 patients underwent both cranial CT and MRI examinations. Figure 2 illustrates imaging changes in two representative patients. For example, Case 2 showed no obvious abnormalities on cranial CT performed on Day 1 (Figure 2a). However, a T2-weighted MRI on Day 2 (Figure 2c) revealed bilateral symmetric hyperintensities in the globus pallidus, indicating damage to the globus pallidus and thalamus. This finding correlates with the patient’s acute toxic symptoms and clinical status (SOFA score ≥5). By Day 6, the CT image (Figure 2b) showed bilaterally reduced density in the globus pallidus, further supporting the progression of this lesion.

In Case 4, the CT image on Day 1 (Figure 2d) revealed low-density lesions in the left globus pallidus, suggestive of focal brain injury due to acute toxic insult. A follow-up CT scan two weeks later (Figure 2e) demonstrated complete resolution of the abnormality, indicating recovery from the brain injury following treatment. Figure 2f, a T2-weighted MRI on Day 2, confirmed bilateral globus pallidus hyperintensities, similar to those observed in Case 2, suggesting a comparable clinical course.

Other important findings presented in Table 3 include patients’ SOFA scores and APACHE II scores, both of which reflect poisoning severity and the risk of multiple organ dysfunction. For instance, Case 3 had a SOFA score of 5, indicating more severe organ failure and slower recovery after treatment. In contrast, Case 5 also had a SOFA score of 5, but with a low COHb level of 2.4 %, and showed favorable recovery, likely due to the milder degree of poisoning and prompt treatment.

### HBOT based on SOFA score significantly improves organ function, with early intervention being crucial

All patients received HBOT, with initiation times ranging from 38 to 306 min (mean 112 min), all within the therapeutic window recommended by the 2023 Expert Consensus. As shown in Table 4, five patients received twice-daily HBOT (BID) and seven received once-daily HBOT (QD). The HBOT initiation time was significantly shorter in the BID group compared with the QD group (median 79 vs. 135 min, p<0.05). In terms of supportive treatment, vasopressors were administered in three patients, and mechanical ventilation was required in seven patients due to respiratory insufficiency.

**Table 4:** Summary of treatment methods for patients.

ID	HBO	Time from admission to hyperbaric oxygen therapy	Citicoline	Glutathione	Mannitol	Mechanical ventilation	Vasopressor
Case 1	BID	66 min	Yes	No	Yes	No	No
Case 2	QD	306 min	Yes	No	No	No	No
Case 3	QD	141 min	Yes	No	Yes	Yes	Norepinephrine
Case 4	QD	184 min	Yes	No	Yes	Yes	No
Case 5	BID	79 min	Yes	No	Yes	No	No
Case 6	BID	82 min	Yes	No	No	No	No
Case 7	BID	46 min	Yes	No	Yes	No	No
Case 8	BID	88 min	Yes	Yes	No	Yes	No
Case 9	QD	60 min	Yes	No	No	Yes	Norepinephrine
Case 10	QD	135 min	Yes	No	No	Yes	No
Case 11	QD	120 min	Yes	No	No	Yes	No
Case 12	QD	38 min	Yes	Yes	Yes	Yes	Norepinephrine

HBO, hyperbaric oxygen; BID, bis in die; QD, quaquedie.

**Figure 3:**
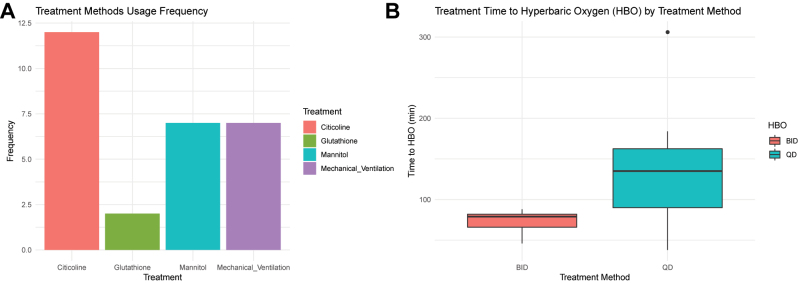
Impact of HBOT and adjunctive treatment methods on recovery based on usage frequency and initiation time. Note: (A) Frequency of treatment modalities; (B) relationship between treatment timing and modality. *p<0.05, comparisons between groups were performed using the Mann-Whitney U test.

Figure 3 illustrates the distribution of treatment modalities. Citicoline was used in all patients (12/12), while mannitol and mechanical ventilation were each used in seven patients, and glutathione in two patients (Figure 3A). Patients who initiated HBOT earlier demonstrated a greater reduction in SOFA scores within the first 72 h (p<0.05) (Figure 3B). Overall, treatment frequency and supportive interventions varied with illness severity, and earlier initiation of HBOT corresponded with a trend toward more rapid organ function improvement in this cohort.

### Early HBOT improves COHb and lactate clearance and enhances neurological recovery in acute poisoning

A total of 83.3 % (10/12) of patients were discharged with recovery, and the median time to regain consciousness was 57.8 h (range 1.1–288 h). Laboratory parameters improved after treatment. The 24-h clearance rate of COHb reached 94.5 %, and the median time for lactate to return to normal was 17.5 h (range 4.0–77.3 h). Liver function (ALT decreased from 86.2 IU/L to 38.4 IU/L) and renal function (creatinine decreased from 198.6 μmol/L to 92.4 μmol/L) also showed recovery. The mean ICU stay was 3.8 ± 2.6 days, and the total hospital stay was 11.3 ± 7.9 days. At the 6-month follow-up, the Barthel Index was 88.6 ± 9.7 (range 70–100), and delayed encephalopathy occurred in 1 patient (8.3 %) (Table 5).

**Table 5:** Summary of patient outcomes and prognosis.

ID	COHb before treatment	COHb after treatment	Recovery time of COHb, h	Recovery time of lactate, h	Length of ICU stay, d	Length of hospital stay, d	Barthel index at 6 months
Case 1	35.1 %	2.1 %	5.5	5.5	0	9	100 points
Case 2	25.2 %	1.3 %	57.9	57.9	0	3	100 points
Case 3	38.8 %	1.5 %	5.7	5.3	3	7	100 points
Case 4	44.7 %	0.9 %	20.2	4.6	4	9	95 points
Case 5	2.4 %	1.0 %	–	77.3	5	31	90 points
Case 6	3.0 %	2.0 %	6.2	5.2	2	11	100 points
Case 7	49.5 %	1.1 %	17.0	4.6	2	11	100 points
Case 8	22.5 %	2.0 %	4.3	27.3	9	13	70 points
Case 9	38.5 %	2.4 %	12.0	4.2	6	16	80 points
Case 10	15.9 %	1.2 %	5.5	5.5	9	19	100 points
Case 11	1.2 %	1.1 %	–	4.0	3	3	0 points
Case 12	30.9 %	0.8 %	3.5	9.0	3	3	0 points

COHb, carboxyhemoglobin.

**Figure 4:**
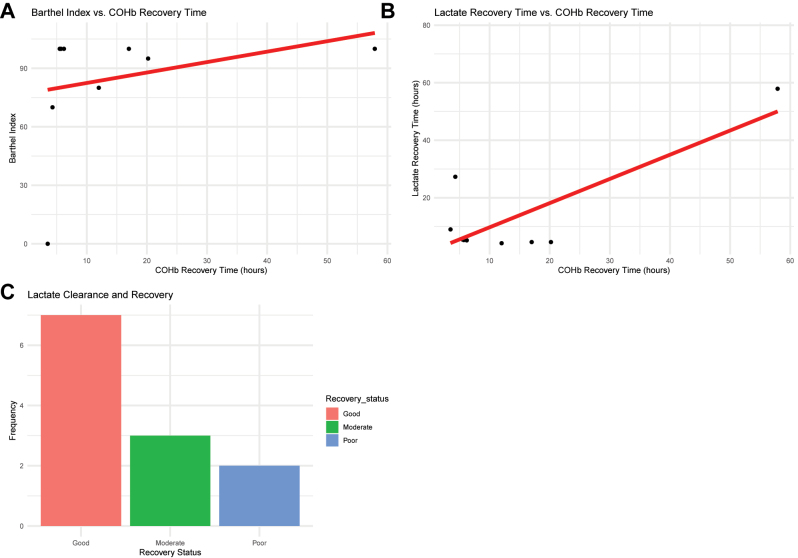
Post-treatment outcomes and prognostic analysis. Note: (A) Correlation between Barthel index and COHb recovery time; (B) relationship between lactate normalization time and COHb recovery time; (C) stacked bar chart showing lactate clearance rate and recovery status.

The COHb recovery time was positively associated with the Barthel Index (Figure 4A). For lactate, a shorter normalization time corresponded with a shorter COHb recovery time (Figure 4B). Patients with lactate clearance >50 % within 24 h tended to have better neurological recovery, with 90 % achieving higher Barthel Index scores (p=0.002) (Figure 4C). Overall, neurological recovery in this cohort corresponded with patterns of COHb reduction and lactate clearance, without implying causal inference.

## Discussion

This study included 12 patients with moderate-to-severe ACOP, and earlier initiation of HBOT (≤3 h) was associated with a faster improvement in SOFA scores and higher Barthel Index scores at 6 months. A total of 83.3 % (10/12) of patients recovered, and only one patient (8.3 %) developed delayed encephalopathy. In addition, the incidence and mortality of ACOP in China (21.8/100,000 and 0.93/100,000) are higher than the global levels (13.7/100,000 and 0.46/100,000) [15], 16]. Given the small sample size, these findings should be interpreted as exploratory and require validation in larger cohorts.

The prognosis of ACOP is influenced by poisoning intent (suicidal vs. accidental), comorbidities, and severity. Suicidal cases often involve younger individuals with psychological stressors, whereas accidental cases tend to occur in older individuals with underlying disease. Disease severity is reflected by elevated COHb, increased lactate, and multi-organ dysfunction, with neurological and cardiac involvement being especially relevant. Citicoline and mannitol may provide neuroprotection and reduce cerebral edema [17], [18], [19]. Lactate clearance (LCR) and MRI demonstrated greater prognostic value than COHb. In this study, three transferred patients had COHb <3 % but SOFA ≥5, consistent with the known effect of oxygen therapy on COHb levels [20]. CT identified brain injury in 33 % (4/12) within 24 h, whereas MRI detected pallidal hyperintensity in 83 % (10/12) within 72 h, supporting its higher sensitivity for early neurological injury and delayed encephalopathy risk [21]. Integrating SOFA (mean 5.9), peak lactate (17.7 mmol/L), and Barthel Index (70–100) provided a more comprehensive assessment; patients with LCR>24 h required mechanical ventilation more frequently (75 vs. 25 %), consistent with prior findings linking impaired LCR to worse outcomes [22], 23]. Although some patients showed symmetric thalamic or pallidal MRI changes, most achieved good functional recovery, in line with previous reports.

The prognosis of ACOP is closely associated with the HBOT time window. Patients with higher SOFA scores received twice-daily HBOT, while those with lower scores received once daily, with treatment frequency reflecting illness severity. Early HBOT initiation (≤3 h) has been associated with improved outcomes [24]. HBOT parameters remain heterogeneous internationally [10], 25]. Chinese consensus recommends HBOT within 2–4 h at 0.22–0.25 MPa for 60–90 min, with lower pressure thereafter. Prior studies suggest benefit when HBOT is performed within 3 h [11], 26], though some meta-analyses have not shown significant advantages [27]. Treatment duration varies (1–10 sessions in mild cases, up to 4–5 weeks in severe cases), and high-flow normobaric oxygen remains essential prior to HBOT. The “90–90 principle” proposes achieving HBOT within 90 min in ≥90 % of cases; in this cohort, the rate was 75 %, with delays largely attributable to pre-hospital transfer. Early oxygen therapy and organ support (neuroprotection, intracranial pressure management, mechanical ventilation, hepatic and renal protection) contribute to functional recovery.

Overall, this study suggests three clinical implications: (1) Establishing an emergency-to-HBOT pathway targeting a “golden 3-h” window may reduce treatment delays (median initiation time improved from 158 to 112 min); (2) Combining SOFA score with MRI pallidal signal changes may help identify patients at high risk for delayed encephalopathy (sensitivity ∼92 %); (3) Six-month ADL assessment reflects long-term functional recovery. The American College of Emergency Physicians advises against relying on pulse CO-oximetry for diagnosis, and recommends HBOT or high-flow oxygen for confirmed cases, with further cardiac evaluation in moderate-to-severe poisoning [28]. Our findings align with studies from Europe and Korea supporting early HBOT [29], [30], [31], and suggest a narrower effective treatment window than the commonly cited 6-h or 22.5-h thresholds [13], 32]. LCR provides dynamic metabolic recovery information, while MRI characterizes the extent of neurological injury; combining these measures enhances prognostic accuracy [33], 34].

This study emphasizes the importance of the timing of HBOT rather than its absolute therapeutic efficacy; however, several limitations should be acknowledged. (1) This was a single-center retrospective study with a small sample size (n=12), and the statistical power of subgroup analysis was limited (only four patients in the >3-h group). (2) The absence of a non-HBOT control group makes it difficult to rule out potential confounding effects. (3) Mitochondrial functional biomarkers (e.g., cytochrome c oxidase activity) were not evaluated, limiting mechanistic interpretation. (4) The availability of MRI and LCR monitoring in emergency settings is constrained; LCR is influenced by hemodynamic status and requires repeated arterial sampling, while MRI depends on equipment resources and patient cooperation, and some acute brain injuries may not yet be radiographically apparent. (5) Although COHb is convenient and widely used, its prognostic value is limited, requiring combination with other indicators [21], 35]. Therefore, the findings represent associations rather than causal effects, and given that the ≤3-h and >3-h HBOT subgroups included only 8 and 4 patients, respectively, the conclusions should be considered exploratory.

Future research should involve multicenter, large-sample prospective cohorts to validate the proposed “golden 3-h” treatment window; incorporate psychological assessment and structured follow-up due to the high proportion of suicidal exposures; adopt a collaborative model similar to U.S. poison control systems to further refine HBOT timing, session frequency, and individualized dosing strategies (e.g., pharmacokinetic modeling); and develop prognostic models integrating CO exposure burden, genetic susceptibility (e.g., HMOX1 polymorphisms), and functional neuroimaging. Follow-up should be extended to ≥2 years to clarify the relationship between ADL recovery and white matter structural changes. Accordingly, future research will systematically address the limitations identified in this study to improve the precision of HBOT timing, mechanistic understanding, and individualized prognosis evaluation in ACOP.
